# Supplementary material for: Dissecting the immunosuppressive tumor microenvironments in Glioblastoma-on-a-Chip for optimized PD-1 immunotherapy
Source: eLife. 2020 Sep 10;9:e52253. doi: 10.7554/eLife.52253 (PMC7556869; doi:10.7554/eLife.52253)
Supplement: Supplementary file 1. [file elife-52253-supp1.docx]

Supplementary Information for

Dissecting the Immunosuppressive Tumor Microenvironments in Glioblastoma-on-a-Chip for Optimized PD-1 Immunotherapy

Xin Cui^†^, Chao Ma^†^, Varshini Vasudevaraja, Jonathan Serrano, Jie Tong, Yansong Peng, Joshua Frenster, Michael Delorenzo, Guomiao Shen, Renee-Tyler Tan Morales, Weiyi Qian, Aristotelis Tsirigos, Andrew S. Chi, Rajan Jain, Sylvia C. Kurz, Erik P. Sulman, Dimitris G. Placantonakis, Matija Snuderl, Weiqiang Chen^*^

^†^ These authors contributed equally to this work

*Correspondence should be addressed to W. Chen (email: wchen@nyu.edu)

**Supplementary File 1A.** **Summary of** **patient-derived GBM cell lines of different molecular subtypes**.

| **Cell line** | **DNA Methylation / RNA Expression Subtype** | **IDH1/2 status by next-generation sequencing** | **MGMT**  **methylation** | **Gender** |
| --- | --- | --- | --- | --- |
| **GBML20** | RTKI/Proneural | Wild-type | Not methylated | M |
| **GBML109** | RTKI/Proneural | Wild-type | Not methylated | F |
| **GS7-11** | RTKI/Proneural | Wild-type | Methylated | F |
| **GBML08** | RTKII/Classic | Wild-type | Methylated | M |
| **GBML83** | RTKII/Classic | Wild-type | Methylated | M |
| **GBML107** | RTKII/Classic | Wild-type | Methylated | F |
| **GBML91** | Mesenchymal | Wild-type | Methylated | M |
| **GSC20** | Mesenchymal | Wild-type | Indetermined | M |
| **GSC289** | Mesenchymal | Wild-type | Unmethylated | M |
| **MGG152** | Astrocytoma IDH mut/ Proneural | Mutated (IDH1) | Methylated | M |

**Supplementary File 1B. Summary of antibodies used in immunofluorescence staining**.

| **Antibody** | **Host** | **Dilution** | **Catalog No.** | **Vender** |
| --- | --- | --- | --- | --- |
| **CD8** | Mouse | 1/50 | 344726 | BioLegend |
| **CD68** | Mouse | 1/50 | 333819 | BioLegend |
| **CD69** | Mouse | 1/50 | 310904 | BioLegend |
| **CD154** | Mouse | 1/50 | 310805 | BioLegend |
| **CD163** | Mouse | 1/50 | 333602 | BioLegend |
| **PD-1** | Mouse | 1/100 | 367402 | BioLegend |
| **PD-L1** | Mouse | 1/100 | MAB1561 | R&D Systems |
| **Perforin** | Mouse | 1/50 | 308106 | BioLegend |
| **Granzyme B** | Mouse | 1/50 | 372208 | BioLegend |
| **Laminin** | Rabbit | 1/50 | PA522901 | Thermo Fisher Scientific |
| **Fibronectin** | Mouse | 1/50 | IC1918P | R&D Systems |
| **Collagen IV** | Mouse | 1/50 | 51-9871-80 | Thermo Fisher Scientific |
| **HA Binding Protein** | Bovine | 1/50 | 385911 | Millipore Sigma |
